# An Epidemiological Study on the Prevalence of the Clinical Features of SARS-CoV-2 Infection in Romanian People

**DOI:** 10.3390/ijerph17145082

**Published:** 2020-07-14

**Authors:** Constantin Ciucurel, Elena Ioana Iconaru

**Affiliations:** Department of Medical Assistance and Physical Therapy, University of Pitesti, 110040 Pitesti, Romania; constantin.ciucurel@upit.ro

**Keywords:** COVID-19, prevalence study, online survey, Romanian population

## Abstract

The aim of this study was to investigate the prevalence of the clinical features of the SARS-CoV-2 infection in Romanian population through a novel online survey. The survey included categorical socio-demographic and health-related variables. A total of 1830 participants were selected for statistical data processing (a response rate of 90.9%). We determined reasonable reliability of the survey section for clinical features of SARS-CoV-2 infection (Cronbach’s Alpha 0.671). Two meaningful dimensions were identified through CATPCA (Categorical Principal Component Analysis) for the survey’s items. We separated two significant clusters of items, each measuring a distinct factor: the sociodemographic characteristics linked to social distancing and the relevant clinical features of SARS-CoV-2 infection. Next, a two-step cluster analysis helped to classify the sample group taking into consideration the similarity of subjects. The clustering revealed a three-cluster solution, with significant differences between clusters and allowed the cluster detection of a group of individuals, possibly more affected by the infection with the SARS-CoV-2 virus. Through binomial logistic regression analysis, we identified a statistically significant prediction model for the presumptive diagnostic of some relevant clinical features of SARS-CoV-2 infection. Our study validated a cost-effective model for rapid assessment of the health status of subjects, adapted to the context of SARS-CoV-2 pandemic.

## 1. Introduction

In the recent past large increases in COVID-19 cases and deaths continue to be reported worldwide, including the EU/EEA countries [1]. The WHO defined the pandemy of COVID-19 as a public health emergency, with serious worldwide consequences in the short, medium, and long term [2]. This pandemic already has a major impact on human communities, through the important effects on the health of individuals, through the overloading of health care services, but also through the complex effects induced by the measures of the social distancing of the population. In this context, researches focused on epidemiological issues are of real use, bringing new opportunities and perspectives for public health policies.

In Romania, the first confirmed SARS-CoV-2 virus infection was recorded on 26 February 2020, with 1–2 cases confirmed daily in the following days. From 10 March, the trend became ascending and the number of cases reached an average of 300 cases/day at the beginning of April [3]. At the time of conducting the research, the trend in Romania was towards an increase in the number of confirmed people through detection of viral RNA by PCR test. Compared to other EU countries, in Romania, the number of people infected with the virus is lower, maybe due to the low number of tests performed. Another factor to mention is the early adoption of social isolation measures by the population and the establishment of the state of emergency for one month by the authorities on 16 March 2020.

Initially, a large part of all confirmed cases was recorded as a result of close contact with infected persons arriving in Romania from abroad (about 300,000 people in 6 weeks from the beginning of the epidemic in Romania), or with their contacts. Subsequently, the share of community transmission increased in that period.

The complexity of the clinical manifestations of SARS-CoV-2 infection is the subject of numerous debates in the scientific world. In this sense, there are several standardization approaches. Mainly, the screening diagnosis is supported by the patient’s epidemiological history (travel/residence in affected areas and/or contact with symptomatic persons during the last 14 days) and by clinical symptomatology (acute fever during the last 72 h which cannot be attributed to another etiological diagnosis) [4]. Most recent research indicates that infection with the SARS-CoV-2 virus commonly shows symptoms such as fever, dry or productive cough, dyspnoea, sore throat, myalgia, and fatigue, general weakness, and pain in the context of viral pneumonia [1,5]. The symptoms described may vary depending on the age of the patients. For example, in adults occur, in order of frequency, fever, cough, chest tightness/pain, fatigue, and sore throat. In contrast, children are more often asymptomatic, with fever and cough being reported in relatively few cases [6].

On the other hand, other clinical elements have been proposed as useful for the diagnosis of the disease, such as digestive symptoms: diarrhoea, anorexia, and nausea [7]. In addition, some clinical manifestations, like anosmia and dysgeusia, although minor in appearance, are considered to have a pathognomonic character [8]. It should be noted that an important category of people infected with the SARS-CoV-2 virus is totally asymptomatic, but can transmit the disease, the percentage going up to 78%, according to some authors [9].

In Romania, the diagnosis of suspicion for SARS-CoV-2 infection is established in the following situations:

(1) A patient with an acute respiratory infection, with sudden onset, having met at least one of three clinical criteria (cough, fever, shortness of breath/increased respiratory rate), without another aetiology that fully explains the clinical picture and with international travel history, within 14 days prior to symptom onset.
(2) A patient with an acute respiratory infection, having been in close contact with a confirmed case of COVID-19 within 14 days prior to symptom onset.
(3) A patient with pneumonia or severe acute respiratory infection (SARI), without another aetiology that fully explains the clinical picture.

Instead, a confirmed case is considered to be a person with laboratory confirmation of infection with SARS-CoV-2, regardless of clinical signs and symptoms [10]. Similarly, according to WHO guidelines, laboratory diagnosis of COVID-19 is based on a positive real-time reverse transcription polymerase chain reaction (rRT-PCR) test for the qualitative detection of nucleic acid from SARS-CoV-2 [11].

Regarding the strategy of testing people in Romania for infection with the SARS-CoV-2 virus, the recommendations of the European Centre for Disease Prevention and Control (ECDC) and World Health Organization (WHO) were applied [12]. Thus, in order, the tests are prioritized (only at the physician’s recommendation) for symptomatic persons with international travel history, symptomatic close contacts of confirmed cases, symptomatic medical-sanitary personnel, pneumonia or SARI cases with unspecified aetiology from all hospitals, institutionalized persons with symptoms, patients before the transplant procedure (asymptomatic) and hematopoietic stem cell donors before donation [13].

Worldwide, there have been numerous initiatives to provide support services for communities hard-pressed by the new clashes that have arisen during this difficult time. Therefore, reliable alternative technological solutions have been proposed to aid health professionals. They are mainly based on mobile internet technologies, such as mobile applications or personal-oriented digital platforms that can carry out population screening activities in a very short time. Essentially, these are self-administered questionnaires to interested persons, which allow identifying the symptomatology and the context of infectious risk [14]. These alternative or complementary methods to the classical laboratory test procedures become extremely useful when the testing capacity is exceeded by the very large number of new cases that have occurred, and/or when the logistical resources are limited. Furthermore, through the geographical tracking and mapping of infected people, it is possible to sustain the global fight against the COVID-19 outbreak [15].

## 2. Materials and Methods

### 2.1. Premises and Aim of the Study

In Romania, the frequency of performing the population testing for the SARS-CoV-2 virus is relatively low, but the epidemiological situation seems to be still under control. However, there are certain risks to the short-term evolution of the pandemic, taking into account, for example, the massive tendency of returning people from risk areas. When returning to Romania, these persons are required to quarantine, or isolate for 14 days, at home or in special spaces. That is why it seems plausible that there are a much larger number of infected people than the one found by direct testing. Thus, the purpose of the study was to conduct a cross-sectional, descriptive, epidemiological investigation by administering an online survey regarding the prevalence of the specific symptomatology of the SARS-CoV-2 virus infection among the Romanian population. The present research aimed to contribute to the development of useful information resources, necessary for the management of a new disease, which has many unknowns.

### 2.2. Participants

The study was designed on a sample of the Romanian population. Thus, a number of 2013 people were initially voluntarily recruited into research, by completing the online questionnaire. Of these, 1830 persons were validated for the statistical processing of the data. The criteria for inclusion in the study refer to the current domicile in Romania and completing the questionnaire in full. The study was approved by our institutional ethical committee (registration number 387/31.03.2020) and all participants provided online informed consent to participate in the research.

### 2.3. Data Acquisition

For online data collection, the software PsyToolkit (https://www.psytoolkit.org/) was used [16,17]. Thus, an online survey was developed, that includes two categories of items, with mandatory completion. The first category (10 items) consists of categorical socio-demographic variables of the participants: age, sex, domicile in a certain county in Romania, type of current residence, type of housing, social status, travel abroad in the last month, contact with a confirmed SARS-CoV-2 virus case, the current situation related to the social isolation imposed by law, the average number of contacts with people in the last two weeks. Of the 10 items, nine have closed-ended questions with a single answer and one item contains an open question (another situation in the context of social isolation imposed by law). The second category (22 items) includes variables that circumscribe the participants’ health, from the physical and psychological point of view, and the 13 most relevant clinical features of infection with the SARS-CoV-2 virus. Of the 22 items, 21 are closed-ended questions with a single answer, and one item has a closed-ended question with multiple choice answers (comorbidities). The survey contains one numerical variable (age) and nominal variables converted to numerical dummy values.

In the preamble of the questionnaire, the participants were informed about the storage and use of the data for research purposes, under conditions of confidentiality and anonymity, according to the laws in force. The questionnaire could be completed by each participant or for any other person in his family (child, older people, etc.). The estimated average time to complete the survey was 3–4 min. The questionnaire could be freely distributed through social networks to increase the size of the sample of participants and was accessed through a dedicated link between 2 April 2020, and 7 April 2020.

### 2.4. Outcomes and Statistical Analysis

The answers obtained were imported and statistically processed using IBM SPSS 20.0 software (IBM Corp., Armonk, NY, USA) in terms of descriptive statistics (mean, standard deviation, frequency distribution, multiple response analysis, Cronbach’s alpha test for the internal consistency of the instrument) and inferential statistics (categorical principal component analysis with optimal scaling, two-step cluster analysis, followed by one-way ANOVA and Pearson’s chi-square test, and binomial logistic regression).

## 3. Results

This section may be divided by subheadings. It should provide a concise and precise description of the experimental results, their interpretation as well as the experimental conclusions that can be drawn.

### 3.1. Respondents

From 2013 persons with current domicile in Romania, a number of 1830 participants were selected for statistical data processing (a response rate of 90.9%). Eligibility was based on completing the online questionnaire in full.

### 3.2. Sociodemographic Characteristics of Study Participants

The mean respondent age was 33.54 ± 13.14 years, the age range varied between 4 and 90 years old, with a sex ratio of 0.369 (494 men/1336 women) and most respondents were between 18 and 50 years (85.6%). In terms of territorial distribution (Table 1), most of the participants (62.5%) come from a single district (Arges), in the second place (8.3%) being those from the country’s capital (Bucharest), the rest being distributed relatively evenly across the country. This can be explained by the fact that initially the questionnaire was distributed on social networks, starting from the close acquaintances of the authors, followed by a branched distribution. 

Other data with possible interest for the interpretation of social distancing refer to the fact that most respondents live in urban areas (71.2%), in individual housing (52.8%), are employed (56.4%), did not travel abroad in the last month (97%), did not have contact with a person infected with the SARS-CoV-2 virus (70.7%), are in self-insulation or confined at home (68.2%) and interacted in the last two weeks with an average of 1–5 people (68.1%).

### 3.3. Health Status Characteristics of Study Participants 

Overall (Table 2), most participants were in good or very good health, based on self-assessment (75.5%), had not been tested for the SARS-CoV-2 virus (99.6%), had a mild or moderate level of physical activity in the last two weeks (66%), are non-smokers (72.1%), had no history of chronic illness (81.1%), have not had Influenza or acute respiratory infections in the last 6 months (75%) and have not been vaccinated for Influenza in the last 6 months (91.8%). Among the personal pathological antecedents, the most common are the cardiovascular ones (6.1%). Regarding the presence of symptoms associated with SARS-CoV-2 virus infection in the last 14 days, the following clinical elements were reported more frequently: headache (33.1%), nasal congestion/leakage of nasal secretions (20.8%), sore throat or dry throat sensation (19.4%) and muscle and/or joint pain (14.5%). The rest of the clinical features registered percentages between 0.8–8.8%. Also, most respondents stated that they have always or occasionally wore a face mask outside the home in the last 14 days (77.5%). Finally, from a psychological point of view, most participants had a mild or moderate level of anxiety about the pandemic with SARS-CoV-2 (71.6%).

### 3.4. Multiple Response Analysis

For the dichotomic items that represent the clinical features of SARS-CoV-2 infection, we applied a multiple response analysis to put into evidence people with multiple symptomatology (Table 3). It is observed that, in total, 1016 subjects (55.5%) reported at least one clinical element of those analyzed. 

### 3.5. Reliability of the Survey Section for Clinical Features of SARS-CoV-2 Infection

To assess the internal consistency of the clinical features section of the survey (items h8–h20 from Table 2) we applied the Cronbach’s Alpha. 

The removal of item h9 led to a slight improvement in Cronbach’s alpha. The reason we gave up this item is that it is redundant with the previous item h8, and 49.5% of respondents stated that they had not measured their body temperature in the last two weeks. In addition, in this way, all items considered for specific clinical features of infection with the SARS-CoV-2 virus (h8 and h10–h19) are of binary data type, with yes/no answers. Thus, the Cronbach’s Alpha, based on standardized items, was 0.671, which is considered to be reasonable for a substantial sample [18] and for measuring a single construct with heterogeneous items, when usually the reliability is underestimated [19]. In fact, from Table 4 negligible or low correlations can be observed between the analyzed clinical features. Of note, however, the highest correlations 0.357 between the items h14 and h15, respectively 0.327 between h11 and h17.

### 3.6. Categorical Principal Component Analysis (CATPCA) with Optimal Scaling

We applied this data reduction technique with different numbers of items from our survey to arrive at a meaningful solution for further cluster analysis. This nonlinear method allows reducing numerous observed variables to a number of uncorrelated principal components and the representation of variables as vectors in regard to these components [20].

Based on the “Eigenvalue greater than one” criterion [21], we found two dimensions (components) to be analyzed. Thus, the CATPCA method helped us to reduce the initial set of 32 variables into 17 variables, grouped on two clusters that belong to the socio-demographic profile, respectively to the health profile of the participants (Table 5 and Table 6). The two clusters were distributed into two dimensions by CATPCA, explaining together 31.98% of the total variance.

**Figure 1:**
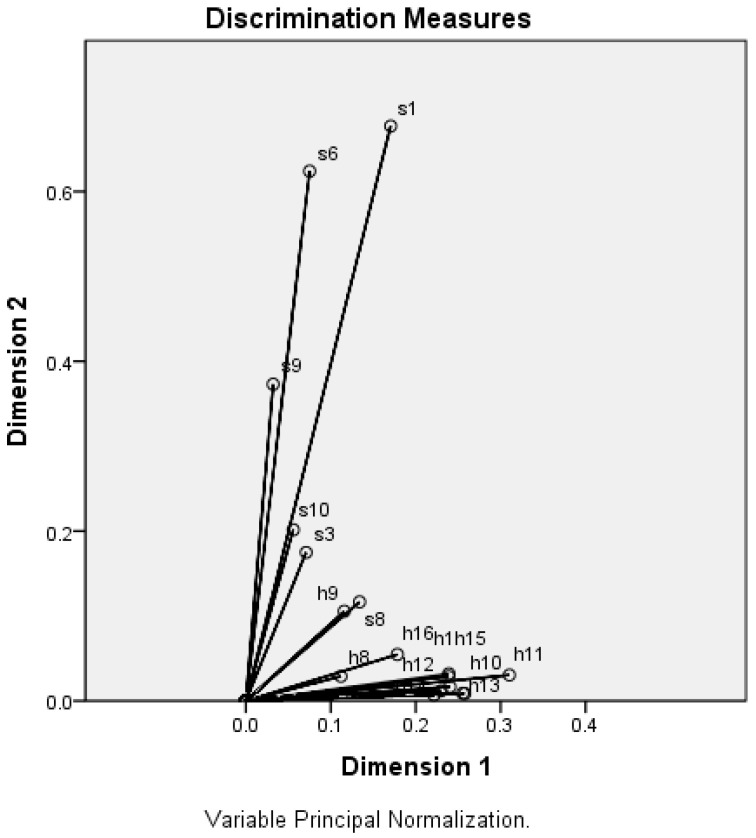
Categorical principal component analysis (CATPCA) biplot—component loadings of the most explanatory variables. Note: items—s1, s3, s6, s8, s9, s10, h1, h8, h9, h10, h11, h12, h13, h14, h15, h16, h17.

In Figure 1 we can observe two groups of correlated vectors pointing in the same direction, and the length of the vectors indicated the most dominant variables (s1, s6, s9, and respectively h11, h13, h10, h15, h17, h1, h12, h14). As a conclusion, CATPCA has categorized the balance of 17 variables into two different dimensions for the sociodemographic profile and the health status. Both dimensions are almost equally important because account for about as much variance (17.27% and 14.71%) and this reason must be interpreted together to describe different areas [22]. Also, all variables have a positive component loading in both dimensions, which means that there is a common factor that correlates positively with all of the variables for each dimension.

### 3.7. Two-Step Clustering Analysis

The two-step clustering analysis with the most dominant variables selected through CATPCA revealed a three-cluster solution (Table 7), with a “silhouette measure of cohesion and separation” of 0.25, indicating a fair cluster solution [23]. The variables in Table 6 are hierarchized from top to bottom, from most to less discriminative between clusters. Statistical differences between clusters were compared using Pearson’s Chi-square test for the categorical variables (s6, s9, h1, h10, h11, h12, h13, h14, h15, h17) and one-way ANOVA for the continuous variables (s1–age). For s1 item (age), the mean value is displayed, and for the categorical items, the percent (%) of records is displayed. All variables presented statistically significant differences in regard to the three clusters (*p* < 0.001).

The three relevant clusters obtained were the following:

(1) Cluster 1 (*n* = 491): participants mostly pupils/students (81.5%), young (mean age 22.77 years), with excellent or very good health (83.3%), self-insulated or confined at home (85.7%), who present in a very small proportion the analysed clinical features (between 0–1.2%).
(2) Cluster 2 (*n* = 619): participants mostly employed (58.2%), mostly young adults (mean age 33.38 years), in very good or good health (79.6%), self-insulated or confined at home (68.8%), who present in a higher proportion the analysed clinical features (1.4–57.4%).
(3) Cluster 3 (*n* = 720): participants mostly employed (83.6%), mostly adults (mean age 41.01 years), with excellent or very good health (65.3), heterogeneous in terms of social isolation, who present in very small proportion the analysed clinical features (0–2.2%).

### 3.8. Binomial Logistic Regression Analysis

A binomial logistic regression (BLR) was repeatedly performed to ascertain the effects of sociodemographic and health characteristics on the likelihood that participants have the clinical features of SARS-CoV-2 infection. After checking the required assumptions, we run the BLR for each clinical feature item (variables with dichtotomous scale, h8, and h10–h20 items) as the dependent variable, and a model with 11 selected items as independent variables (based on the principle of avoiding multicollinearity): s1, s2, s4, s5, s7, h2, h4, h5, h6, h7, and h21. The logistic regression model was statistically significant (*p* < 0.001) for h10–h19 items (Table 8). 

Table 8 shows the percentages of variance (Nagelkerke R^2^) that can be attributed to the investigated regression models and the Odds Ratios (OR) for each variable with statistically significant influence in the regression model. These results (Table 8) will be commented on in the Discussion section.

## 4. Discussion

The present study did not set as its objective the diagnosis of people infected with the SARS-CoV-2 virus, but only the identification of the presence of clinical features that are specific to this disease among the population. The subject is very topical in light of current discussions about the pandemic trend, the movement restrictions imposed at the community level, but also the limited possibilities of mass testing. The identification of risk groups is essential for the subsequent efficient application of the measures of diagnostic, isolation, quarantine, treatment, etc. of the selected cases.

First of all, from this study a series of descriptive informational elements can be detached, which can be analyzed and interpreted in the specific Romanian situational context (for example, the percentage of smokers, the level of Influenza vaccination of the population, the respondent’s level of physical activity, the psychological impact of the pandemic, etc.). It should be mentioned that at the time of completing the data gathering in Romania, 4417 cases of SARS-CoV-2 virus infection were confirmed, 196 deaths were caused by this disease, and about 45,000 diagnostic rRT-PCR tests were performed [24]. 

Within the two weeks considered, we identified single or associated clinical features that are part of the diagnostic criteria of SARS-CoV-2 virus infection, in a significant percentage of participants (55.5%). In other words, the percentage of detection of clinical elements among the studied sample varied between 33.1% for headache and 0.8% for loss of taste and/or odour. Not all individuals are carriers of the virus, the symptoms of this disease being diverse [25] and largely nonspecific, mainly associated with respiratory and few extrapulmonary signs [26]. On average, subjects who reported symptoms had 2.25 clinical features per individual. The highest correlation recorded (but of low intensity) was between chest pain or chest pressure sensation and dyspnoea (breathing difficulty), suffocation, and/or shortness of breath, respectively between increased fatigue and muscle and/or joint pain. Our results suggest the presence of possible acute respiratory infections among respondents, which may have various aetiologies. Given the present epidemiological situation, we cannot exclude, in a part of the study sample, the variant of infection with the SARS-CoV-2 virus, or the variant of infection or co-infection with other respiratory viruses. The underdiagnoses of SARS-CoV-2 virus infection in the context of respiratory viral coinfections [27], as well as the frequent spread in the population of asymptomatic, presymptomatic or paucisymptomatic forms of the disease, remains a matter of debate [9,28]. However, in the case of Romania, paradoxically, the number of people infected with the SARS-CoV-2 virus and the consecutive deaths were well below the European average at the beginning of the pandemic, a situation that can have multiple explanations. Nevertheless, it remains open the hypothesis according to which the disease may be far more frequent among people of Romania and its prevalence may be underestimated, similar to other countries [29].

The section of our survey for clinical features assessment of infection with SARS-CoV-2 had an acceptable internal consistency. Since we are talking about a condition with a high polymorphism, in which most individuals are asymptomatic or have mild forms [30], the instrument appears to be reliable for early digital health screening, and also easy to be implemented.

Following the application of CATPCA as data reduction technique for the entire survey, the outcomes of the categorized variables showed that we can use only 17 items to investigate the multifaceted profile of the sample group, related to the infection with SARS-CoV-2 virus. Practically, we succeeded to classify the variables into two meaningful dimensions and the total variance is explained for the principal components solution to a percentage of 31.98%. The CATPCA results mapped the nature of the interaction between the sociodemographic profile and the health status of the respondents and therefore we validated the proposed categorization attempt for classification procedure.

Next, we introduced the CATPCA results in a two-step cluster analysis and we reported a model with three meaningful classificatory clusters, statistically different in regard to the variables considered (*p* < 0.001). Differentiating the three clusters is important because it offers the possibility of separating a risk group for the disease. Thus, the subjects from the second cluster were the most clinically affected, with a heterogeneity of symptoms, but without presenting serious clinical forms of the disease. Identifying this cluster becomes useful because it includes people at higher risk of actually being infected with the SARS-CoV-2 virus. In contrast, the other two clusters (one for young people and one for adults) stand out through a better state of health and almost no clinical manifestations. The hierarchical distribution of clinical features on clusters according to their predictive value is also interesting, the order being h13, h17, h11, h12, h14, h15, h10, different from the one resulting from the CATPCA analysis, according to variance. From the same point of view, prominent hierarchical positions have the social status (s6) and age (s1 item), as determinants of the sociodemographic profile of the subjects. 

Most of the symptoms described are superimposable with the major clinical manifestations in all coronavirus infections, including the one with SARS-CoV-2 virus (fever, chills, cough, shortness of breath, generalized myalgia, malaise, drowsy, diarrhoea, confusion, dyspnoea, and pneumonia [25]. Multiple studies report the prevalent clinical manifestations for SARS-CoV-2 infection like fever, cough, dyspnoea, with a fever frequency significantly higher in adults compared to children [6,26,31]. In our study, the second cluster presented the most important fields in estimating the model, based on the predictor importance, in order: the social status, sore throat or dry throat sensation, age, muscle and/or joint pain, and increased fatigue.

It should be mentioned the absence of fever in the last 14 days as an important emerging variable in CATPCA analysis, which is why it was not included in the two-step cluster analysis. This can be correlated with the fact that 49.5% of the subjects stated that they did not measure their temperature during the considered period. Moreover, it has been shown that the correct self-perception of fever is achieved in about 80% of cases if the body temperature is higher than 38 °C [32]. We can plausibly consider that the subfebrile states were not noticed by the respondents and/or the fever does not represent a significant feature for the mild forms of registered respiratory infections. In this regard we recall the results of recent research that estimate that a significant part of people tested positive for SARS-CoV-2 virus infection, with potential for disease transmission, are asymptomatic or have a mild illness, with low or absent fever [33]. Yet another study claims that among people confirmed positive for SARS-CoV-2 virus, fever was uncommon (0.7%), and most individuals (87.8%) were asymptomatic [34].

The results must be interpreted taking into account the testing strategy for SARS-CoV-2 virus in Romania, which implies a prioritization of the subjects according to the medical context, epidemiological investigations, and also the available resources. Even in the most developed countries, the rRT-PCR testing is applied in selected cases, due to the low availability of key supplies [35]. The situation in Romania is also comparable to that of many European countries, which have a similar testing strategy, based on WHO recommendations [36].

In the final stage of the research, a binomial logistic model was tested to establish the relationship between the likelihood that the clinical features of the infection with SARS-CoV-2 are related to the selected predictor variables. The results presented in Table 8 revealed that some predictors are significantly associated with most clinical features (items h10–h19). The most relevant associations according to the proposed regression model refer to predictor items h4, h5, h6, and h21 and s1. Thus, the odds of having some clinical features were greater for smokers, for people with chronic diseases, and people with Influenza or acute respiratory infections in the last 6 months. Also, the systematic face mask wearing outside the home represents a protection factor against the development of clinical features, and increasing age was associated with a higher likelihood of exhibiting some clinical features. However, the Nagelkerke R^2^ values were relatively small, even the logistic regression models were statistically significant. In other words, the contribution of the mentioned explanatory variables in the prediction of clinical features is statistically significant, but the interpretation must be made with caution because the effect size is small. 

Other studies confirm smoking as a risk factor for infection with the SARS-CoV-2 virus, causing more severe forms of the disease [37,38]. In the same sense of interpretation, it has been shown that the infection with the SARS-CoV-2 virus is more frequent in patients with chronic medical conditions [31,39] and with a higher lethality [40]. Our results also indicated the additional risk of presenting clinical symptoms after an episode of Influenza or respiratory acute infection in the last 6 months. Interestingly, the chronic respiratory infections appear to be under-represented in the comorbidities reported for patients with COVID-19, possibly due to an altered immune response or therapies used in these cases [41]. Instead, our data suggest that the status of post-acute respiratory infection increases the risk for respiratory reinfection, possibly with the SARS-CoV-2 virus.

Another notable result of our study was the finding of the protective effect of constant wearing a protective mask when moving out of the home. Thus, this fact has been revealed in meta-analyses that have investigated the effectiveness of these physical methods, as simple and low-cost interventions, to reduce respiratory virus infections [42,43]. Finally, our results confirmed that the risk of presenting the investigated clinical symptoms increases proportionally with age, the young ages being generally spared, as other authors stated [6,44,45].

Overall, we proved the practical usefulness of a survey of self-reporting tracking of symptoms of the SARS-CoV-2 virus. At the moment there is a special interest in developing such web-designed applications and researchers are looking for some predictive models based on clinical symptoms of the disease, which can contribute to target screening strategies [46]. In conclusion, through the statistical approach, we managed to validate the key elements of a survey that can identify a risk group for presenting the specific clinical features of SARS-CoV-2 virus infection, during social distancing imposed by the pandemic situation. Further testing of people at risk for infection will allow the detection of real cases, which require a complex therapeutic approach. Nevertheless, the administration of the questionnaire also has educational and formative values as it helps respondents to acquire knowledge on the clinical manifestations of the SARS-CoV-2 infection. In this way, they can better understand the disease and the importance of self-management of healthy behaviors.

### Limitations and Strengths of the Study

One limitation of the study derives from the heterogeneous distribution of the subjects’ age, sex, and territory of residence. This is explained by the fact that the survey was distributed on the web according to the model of social network circles, starting from the authors of the article. Also, it remains to be discussed, as in any study of this type, the honesty of the respondents in conditions of anonymity, as well as their subjectivism in selecting the appropriate answers. Another limitation of the study refers to the low proportion of older adults in the sample, this age group being the most affected by SARS-CoV-2 infection. Instead, the large number of respondents, the accessibility of the questions in terms of content, the very short time to complete the questionnaire, the rapid construction of the database, and the complex statistical analysis of the data proved to be strengths of the research.

## 5. Conclusions

The present study validated a cost-effective model for rapid assessment of the health status of the Romanian population in the form of an online survey, adapted to the existential circumstances of the SARS-CoV-2 virus pandemic. We determined a reasonable reliability of the survey section for clinical features of SARS-CoV-2 infection (Cronbach’s Alpha 0.671). The CATPCA analysis followed by a two-step cluster analysis revealed a three-cluster solution, with significant differences between clusters (*p* < 0.001), which allowed the cluster detection of a group of individuals, possibly more affected by the infection with the SARS-CoV-2 virus. Through binomial logistic regression analysis we identified a statistically significant prediction model for the presumptive diagnostic of some relevant clinical features of the infection with the SARS-CoV-2 virus.

Our survey could be easily applied on a large scale, at an institutional level to monitor in real time the health of people facing the consequences of the pandemic. Thus, the approach can support the efforts of making prompt decisions regarding the implementation of health programs, population diagnostic screening, isolation/quarantine of risk groups, and/or hospitalization of serious cases.

## Figures and Tables

**Table 1:** Summary of the sociodemographic characteristics of study participants (N = 1830).

Item Code	Variable	Frequency (*n*)	Percent (%)
s1	Age		
	4–17 years	49	2.7
	18–50 years	1567	85.6
	51–64 years	180	9.8
	65–90 years	34	1.9
s2	Sex		
	Male	494	27
	Female	1336	73
s3	Domicile (42 districts)		
	District 3 (Arges)	1144	62.5
	District 42 (Bucharest)	152	8.3
	Rest of districts	534	29.2
s4	Type of current residence		
	Urban	1303	71.2
	Rural	527	28.8
s5	Type of housing		
	Individual building	966	52.8
	Collective building	864	47.2
s6	Social status		
	Toddler/pre-schooler	3	0.2
	Pupil/Student	583	31.8
	Employee	1033	56.4
	Freelancer	95	5.2
	Unemployed	17	0.9
	Retired	54	3
	Housewife/househusband	45	2.5
s7	Travel abroad in the last month		
	Yes	55	3
	No	1775	97
s8	Contact with a confirmed SARS-CoV-2 case		
	Yes	6	0.3
	No	1294	70.7
	I don’t know	530	29
s9	Current situation related to the social distancing imposed by law		
	Quarantine	69	3.8
	Self-insulated	831	45.4
	Hospitalized	1	0.1
	Confined at home	417	22.8
	In the workplace	253	13.8
	Another situation	259	14.1
s10	Average number of contacts with people in the last two weeks		
	None	165	9
	1–5 people	1246	68.1
	6–10 people	197	10.8
	More than 10 people	222	12.

**Table 2:** Summary of the health status characteristics of study participants (N = 1830).

Item Code	Variable	Frequency (*n*)	Percent (%)
h1	Qualitative self-assessment of the health status		
	Excellent	363	19.8
	Very good	821	44.9
	Good	560	30.6
	Satisfying	75	4.1
	Weak	11	0.6
h2	Testing for SARS-CoV-2 virus		
	Tested positive	0	0
	Tested negative	8	0.4
	Non-tested	1822	99.6
h3	Maximum level of physical activity achieved in the last two weeks		
	Very easy	438	23.9
	Mild	462	25.3
	Moderate	745	40.7
	Vigorous	142	7.8
	Very heavy	22	1.2
	Maximal	21	1.1
h4	Smoking		
	Yes	510	27.9
	No	1320	72.1
h5	Pathological personal history 1—chronic cardiovascular diseases		
	Yes	112	6.1
	No	1718	93.9
	Pathological personal history 2—chronic respiratory diseases		
	Yes	56	3.1
	No	1774	96.9
	Pathological personal history 3—chronic neurological diseases		
	Yes	19	1
	No	1811	99
	Pathological personal history 4—chronic renal diseases		
	Yes	26	1.4
	No	1804	98.6
	Pathological personal history 5—chronic rheumatic diseases		
	Yes	87	4.8
	No	1743	95.2
	Pathological personal history 6—diabetes mellitus		
	Yes	22	1.2
	No	1808	98.8
	Pathological personal history 7—oncological diseases		
	Yes	12	0.7
	No	1818	99.3
	Pathological personal history 8—others		
	Yes	97	5.3
	No	1733	94.7
	Pathological personal history 9—none		
	Without chronic diseases	1484	81.1
	With chronic diseases	346	18.9
h6	Influenza or acute respiratory infections in the last 6 months		
	Yes	457	25
	No	1373	75
h7	Influenza vaccination in the last 6 months		
	Yes	150	8.2
	No	1680	91.8
h8	Clinical feature 1—fever in the last 14 days		
	Yes	21	1.1
	No	1809	98.9
h9	Clinical feature 2—maximum body temperature measured in the last 14 days		
	36.5 °C	684	37.4
	37 °C	186	10.2
	37.5 °C	40	2.2
	38 °C	7	0.4
	38.5 °C	6	0.3
	Non-measured	907	49.5
h10	Clinical feature 3—chills and/or increased perspiration in the last 14 days		
	Yes	61	3.3
	No	1769	96.7
h11	Clinical feature 4—increased fatigue in the last 14 days		
	Yes	161	8,8
	No	1669	91.2
h12	Clinical feature 5—episodes of persistent dry or productive cough in the last 14 days		
	Yes	154	8.4
	No	1676	91.6
h13	Clinical feature 6—sore throat or dry throat sensation in the last 14 days		
	Yes	355	19.4
	No	1475	80.6
h14	Clinical feature 7—chest pain or chest pressure sensation in the last 14 days		
	Yes	99	5.4
	No	1731	94.6
h15	Clinical feature 8—dyspnoea (breathing difficulty), suffocation and/or shortness of breath in the last 14 days		
	Yes	82	4.5
	No	1748	95.5
h16	Clinical feature 9—headache in the last 14 days		
	Yes	606	33.1
	No	1224	66.9
h17	Clinical feature 10—muscle and/or joint pain in the last 14 days		
	Yes	265	14.5
	No	1565	85.5
h18	Clinical feature 11—nasal congestion/leakage of nasal secretions in the last 14 days		
	Yes	381	20.8
	No	1449	79.2
h19	Clinical feature 12—diarrhoea, nausea, loss of appetite and/or vomiting in the last 14 days		
	Yes	93	5.1
	No	1737	94.9
h20	Clinical feature 13—loss of taste and/or odour in the last 14 days		
	Yes	14	0.8
	No	1816	99.2
h21	Face mask wearing outside the home in the last 14 days		
	Always	852	46.6
	Occasionally	566	30.9
	Never	412	22.5
h22	Anxiety level in the context of the pandemic with SARS-CoV-2		
	Not anxious	350	19.1
	Mild anxious	747	40.8
	Moderately anxious	563	30.8
	Very anxious	133	7.3
	Extremely anxious	37	2

**Table 3:** Multiple response analysis, frequencies and percent of cases.

		Responses		Percent of Cases
		*n*	Percent	
Items ^a^	h8	21	0.9%	2.1%
	h10	61	2.7%	6.0%
	h11	161	7.0%	15.8%
	h12	154	6.7%	15.2%
	h13	355	15.5%	34.9%
	h14	99	4.3%	9.7%
	h15	82	3.6%	8.1%
	h16	606	26.4%	59.6%
	h17	265	11.6%	26.1%
	h18	381	16.6%	37.5%
	h19	93	4.1%	9.2%
	h20	14	0.6%	1.4%
Total		2292	100.0%	225.6%

^a^ Dichotomy group tabulated at value 1 (answer yes)

**Table 4:** Inter-item correlation matrix (clinical features of SARS-CoV-2 infection).

	h8	h10	h11	h12	h13	h14	h15	h16	h17	h18	h19	h20
h8	1.000											
h10	0.237	1.000										
h11	0.093	0.265	1.000									
h12	0.078	0.163	0.191	1.000								
h13	0.103	0.186	0.218	0.269	1.000							
h14	0.065	0.144	0.190	0.154	0.176	1.000						
h15	0.125	0.122	0.231	0.248	0.174	0.357	1.000					
h16	0.077	0.141	0.212	0.130	0.254	0.155	0.117	1.000				
h17	0.072	0.174	0.327	0.155	0.195	0.149	0.129	0.212	1.000			
h18	0.058	0.122	0.135	0.252	0.232	0.109	0.091	0.223	0.118	1.000		
h19	0.092	0.096	0.165	0.073	0.144	0.154	0.106	0.154	0.159	0.151	1.000	
h20	−0.009	0.089	0.083	0.109	0.084	0.034	0.042	0.005	0.071	0.109	0.065	1.000

**Table 5:** Model summary for CATPCA.

Dimension	Cronbach’s Alpha	Variance Accounted For		
		Total (Eigenvalue)	Inertia	% of Variance
1	0.701	2.937	0.173	17.277
2	0.638	2.501	0.147	14.710
Total		5.438	0.320	
Mean	0.672 ^a^	2.719	0.160	15.993

^a^ Mean Cronbach’s Alpha is based on the mean eigenvalue.

**Table 6:** Discrimination measures for CATPCA.

	Dimension		Mean
	1	2	
s1	0.171	0.677	0.424
s3	0.071	0.175	0.123
s6	0.075	0.624	0.350
s8	0.134	0.117	0.125
s9	0.032	0.373	0.203
s10	0.056	0.202	0.129
h1	0.239	0.029	0.134
h8	0.112	0.029	0.070
h9	0.116	0.106	0.111
h10	0.255	0.009	0.132
h11	0.310	0.030	0.170
h12	0.230	0.012	0.121
h13	0.258	0.009	0.133
h14	0.221	0.007	0.114
h15	0.240	0.017	0.128
h16	0.178	0.055	0.116
h17	0.239	0.032	0.135
Active Total	2.937	2.501	2.719
% of Variance	17.277	14.710	15.993

**Table 7:** Characteristics of the three clusters identified by two-step clustering with variable selection based on CATPCA.

Item Code	Variable	Cluster 1 *n* = 491	Cluster 2 *n* = 619	Cluster 3 *n* = 720	*p* Value
s6	Social status (%)				*p* < 0.001 ^a^
	Toddler/pre-schooler	0.6%	0	0	
	Pupil/Student	81.5%	29.6%	0	
	Employee	14.5%	58.2%	83.6%	
	Freelance	0	6.1%	7.9%	
	Unemployed	0.6%	0.8%	1.2%	
	Retired	0	2.1%	5.7%	
	Housewife/househusband	2.9%	3.2%	1.5%	
h13	Clinical feature 6—sore throat or dry throat sensation in the last 14 days (%)				*p* < 0.001 ^a^
	Yes	0	57.4%	0	
	No	100%	42.6%	100%	
s1	Age (mean ± SD, years)	22.77 ± 6.45	33.38 ± 12.84	41.01 ± 11.59	*p* < 0.001 ^b^
h17	Clinical feature 10—muscle and/or joint pain in the last 14 days (%)				*p* < 0.001 ^a^
	Yes	1.2%	39.3%	2.2%	
	No	98.8%	60.7%	97.8%	
h11	Clinical feature 4—increased fatigue in the last 14 days (%)				*p* < 0.001 ^a^
	Yes	0.2%	25.5%	0.3%	
	No	99.8%	74.5%	99.7%	
h12	Clinical feature 5—episodes of persistent dry or productive cough in the last 14 days (%)				*p* < 0.001 ^a^
	Yes	1%	23.1%	0.8%	
	No	99%	76.9%	99.2%	
s9	Current situation related to the social distancing imposed by law				*p* < 0.001 ^a^
	Quarantine	7.7%	4.2%	0.7%	
	Self-insulated	50.9%	48%	39.4%	
	Hospitalized	0.2%	0	0	
	Confined at home	34.8%	20.8%	16.2%	
	In the workplace	0.6%	16.3%	20.7%	
	Another situation	5.7%	10.7%	22.9%	
h14	Clinical feature 7—chest pain or chest pressure sensation in the last 14 days (%)				*p* < 0.001 ^a^
	Yes	1.2%	1.4%	0.1%	
	No	98.8%	85.1%	99.9%	
h15	Clinical feature 8—dyspnoea (breathing difficulty), suffocation and/or shortness of breath in the last 14 days (%)				*p* < 0.001 ^a^
	Yes	0.4%	12.9%	0	
	No	99.6%	87.1%	100%	
h1	Qualitative self-assessment of the health status (%)				*p* < 0.001 ^a^
	Excellent	32%	10.8%	19.3%	
	Very good	51.1%	38.6%	46%	
	Good	16.5%	41%	31.2%	
	Satisfying	0.2%	8.4%	3.1%	
	Weak	0.2%	1.1%	0.4%	
h10	Clinical feature 3—chills and/or increased perspiration in the last 14 days (%)				*p* < 0.001 ^a^
	Yes	0%	9.9%	0	
	No	100%	90.1%	100%	

^a^ Pearson’s Chi-square test. ^b^ One-way ANOVA.

**Table 8:** Outputs of binomial logistic regression analysis (N = 1830).

Independent Variable	Omnibus Test of Model Coefficients		Model Summary	Regression Coefficients and Significance Level				
	Chi-Square	*p*-Value	Nagelkerke R Square	Predictor	B	OR = Exp(B)	95% CI for OR	*p*-Value
h10	37.575	0.001	0.080	h6	1.188	3.281	1.942–5.542	0.001
h11	52.059	0.001	0.062	h4	0.573	1.774	1.254–2.509	0.001
				h5 *	0.724	2.062	1.376–3.091	0.001
				h6	0.791	2.205	1.571–3.095	0.001
				h21 (1)	−0.549	0.578	0.382–0.873	0.009
h12	118.563	0.001	0.143	s5	−0.434	0.648	0.428–0.982	0.041
				h4	0.662	1.938	1.348–2.786	0.001
				h5 *	0.706	2.026	1.343–3.058	0.001
				h6	1.554	4.729	3.341–6.693	0.001
h13	43.370	0.001	0.037	h6	0.722	2.059	1.602–2.646	0.001
h14	36.785	0.001	0.058	h4	0.842	2.322	1.522–3.543	0.001
				h5 *	0.621	1.860	1.126–3.072	0.015
				h6	0.574	1.776	1.156–2.728	0.009
				h21 (1)	−0.688	0.502	0.298–0.847	0.010
h15	62.828	0.001	0.110	h4	0.690	1.994	1.238–3.212	0.005
				h5 *	1.334	3.796	2.271–6.343	0.001
				h6	0.954	2.596	1.640–4.109	0.001
				h21 (1)	−0.995	0.370	0.205–0.666	0.001
				h21 (2)	−0.709	0.492	0.282–0.859	0.013
h16	94.766	0.001	0.070	s1	0.027	1.028	1.019–1.037	0.001
				s2	−0.384	0.681	0.538–0.862	0.001
				h4	0.404	1.498	1.203–1.866	0.001
				h5 *	0.299	1.348	1.021–1.780	0.035
				h6	0.424	1.528	1.218–1.915	0.001
h17	58.030	0.001	0.055	h4	0.429	1.536	1.154–2.043	0.003
				h5 *	0.731	2.078	1.499–2.882	0.001
				h6	0.612	1.844	1.392–2.444	0.001
h18	50.597	0.001	0.043	s1	0.11	1.011	1.001–1.021	0.028
				h4	0.294	1.342	1.045–1.724	0.021
				h6	0.601	1.824	1.423–2.336	0.001
				h21 (1)	−0.433	0.649	0.484–0.869	0.004
				h21 (2)	−0.399	0.712	0.544–0.931	0.013
h19	37.646	0.001	0.062	s1	0.040	1.041	1.021–1.062	0.001
				s7	1.020	2.772	1.126–6.823	0.027
				h5 *	0.703	2.020	1.193–3.419	0.009
				h6	0.721	2.057	1.325–3.192	0.001

Note: * subitem h5 (Pathological personal history 9—none); OR—Odds Ratio; *p*—significance level; for all items, the last answer represents the reference category; (1): always wearing face mask; (2): occasionally wearing face mask
